# Concomitant Gastric and Duodenal Metastases From Cervical Cancer Presenting With Upper Gastrointestinal Bleeding: A Rare Case

**DOI:** 10.7759/cureus.84620

**Published:** 2025-05-22

**Authors:** Mohamad Al Ayoubi, Sally H Abi Dargham, Fatima I Hsayan, Maroun H Sadek, Antoine Abi Abboud

**Affiliations:** 1 Gastroenterology, Lebanese University Faculty of Medicine, Beirut, LBN; 2 Internal Medicine, Lebanese University Faculty of Medicine, Beirut, LBN; 3 Hematology and Medical Oncology, Lebanese Hospital Geitaoui-University Medical Center, Lebanese University, Beirut, LBN; 4 Hematology and Medical Oncology, Lebanese University, Beirut, LBN; 5 Gastroenterology and Hepatology, Lebanese Hospital Geitaoui-University Medical Center, Lebanese University, Beirut, LBN; 6 Gastroenterology and Hepatology, Lebanese University, Beirut, LBN

**Keywords:** acute upper gastrointestinal bleed, cervical cancer, cervical squamous cell carcinoma, duodenal metastasis, gastric metastasis

## Abstract

Upper gastrointestinal bleed is a frequent medical emergency, which may arise from multiple pathologies. Metastatic lesions to the stomach and duodenum, although rare, may be an underlying cause. Specifically, metastasis from a primary cervical malignancy is extremely infrequent, with only a few cases reported in the literature. In addition, concomitant metastatic lesions in the stomach and duodenum from cervical cancer have not been reported yet. We present the case of a 46-year-old female patient known to have cervical squamous cell carcinoma, metastatic to the lungs, bone, and liver, presenting for melena and hematemesis. Endoscopy revealed ulcerating mass lesions in the stomach and duodenum, confirmed as metastatic epidermoid carcinoma on pathology.

Hence, metastasis to the stomach and the duodenum should be kept in mind in a patient with cervical cancer presenting with upper gastrointestinal symptoms.

## Introduction

Upper gastrointestinal bleed, defined as bleeding originating proximally to the Treitz ligament, is one of the most frequent medical emergencies necessitating hospital admission. Typical symptoms include hematemesis, coffee-ground emesis, and melena, with hematochezia being less common [1]. The leading cause is peptic ulcer disease, accounting for 35-50% of the cases, most often related to *Helicobacter pylori* infection and the use of non-steroidal anti-inflammatory drugs [2]. Other etiologies are esophageal varices, erosive esophagitis, Mallory-Weiss tear, angiodysplasia, and neoplasm [3]. However, other underlying pathologies such as metastatic tumor are rare.

Cervical cancer is one of the most prevalent malignancies and a leading cause of death in women, ranked fourth worldwide after breast, lung, and colorectal cancer [4]. It primarily presents as squamous cell carcinoma in 80-85% of the cases, caused by carcinogenic strains of human papillomavirus (HPV) in individuals with behavioral and infectious risk factors [5]. Adenocarcinoma accounts for the rest (15-20%) of the cases [6]. Metastasis to distant organs occurs via hematogenous spread, predominantly to the lungs, bone, and liver. In addition, other sites of metastasis include pelvic and para-aortic lymph nodes through lymphatic spread and direct invasion of tumor cells to adjacent organs [7].

Metastasis to the stomach and duodenum from distant primary malignancies is uncommon. Moreover, the presence of metastatic lesions in the stomach and the duodenum concomitantly with a primary cervical squamous cell carcinoma has not been previously reported in the literature. This report highlights a case of upper gastrointestinal bleed found to be originating from metastatic cervical cancer to the stomach and duodenum.

## Case presentation

A 46-year-old female patient, known to have squamous cell carcinoma of the cervix, metastatic to the lungs, bone, and liver, negative for human epidermal growth factor receptor 2 (HER2), negative for microsatellite instability (MSI), and negative for programmed death-ligand 1 receptor (PD-L1), treated by hysterectomy and chemotherapy (cisplatin 40 mg/m^2^ weekly for seven cycles) with radiotherapy concomitantly, presented to the Emergency Department for multiple episodes of bloody vomiting, compatible with hematemesis. The patient also mentioned a three-day history of melena. In addition, she complained of chronic fatigue with nausea and occasional light-headedness. Vital signs on admission were within the normal range. On physical examination, the patient was conscious, cooperative, and oriented. Auscultation of the heart and lungs was unremarkable. The abdomen was soft, with mild diffuse tenderness on palpation. Laboratory studies at presentation showed severe iron deficiency anemia, leukocytosis with neutrophilia, thrombocytopenia, elevated inflammatory markers, acute kidney injury, and elevated liver enzymes (Table 1).

**Table 1:** Key laboratory test results.

Test	Result	Reference Range
Hemoglobin (g/dL)	4.7	12-15.5
Hematocrit (%)	14.9	37-47
Serum iron (mcg/dL)	19	50-170
White blood cells (/mm^3^)	16,010	4,000-11,000
Neutrophils (%)	90	40-60
Platelets (/mm^3^)	107,000	142,000-450,000
C-reactive protein (mg/dL)	58.9	<0.5
Procalcitonin (ng/mL)	1.5	<0.1
Serum creatinine (mg/dL)	2.04	0.72-1.25
Blood urea nitrogen (mg/dL)	49	20-45
Alkaline phosphatase (U/L)	278	44-147
Gamma-glutamyl transferase (U/L)	369	0-50
Serum glutamic oxaloacetic transaminase (U/L)	69	8-45
Serum glutamic pyruvic transaminase (U/L)	48	7-56

Computed tomography (CT) scan of the thorax, abdomen, and pelvis was performed without contrast, followed by multiplanar reconstruction. It showed extensive pulmonary metastasis with right middle lobe infiltrates and hypodense liver lesions, as well as subcutaneous soft tissue mass in the left abdominal wall. In addition, abdominal ultrasound revealed hepatomegaly reaching 20 cm and multiple hepatic lesions with heterogenous echogenicity compatible with metastasis to the liver. However, there was no intrahepatic or extrahepatic biliary tree dilation.

**Figure 1:**
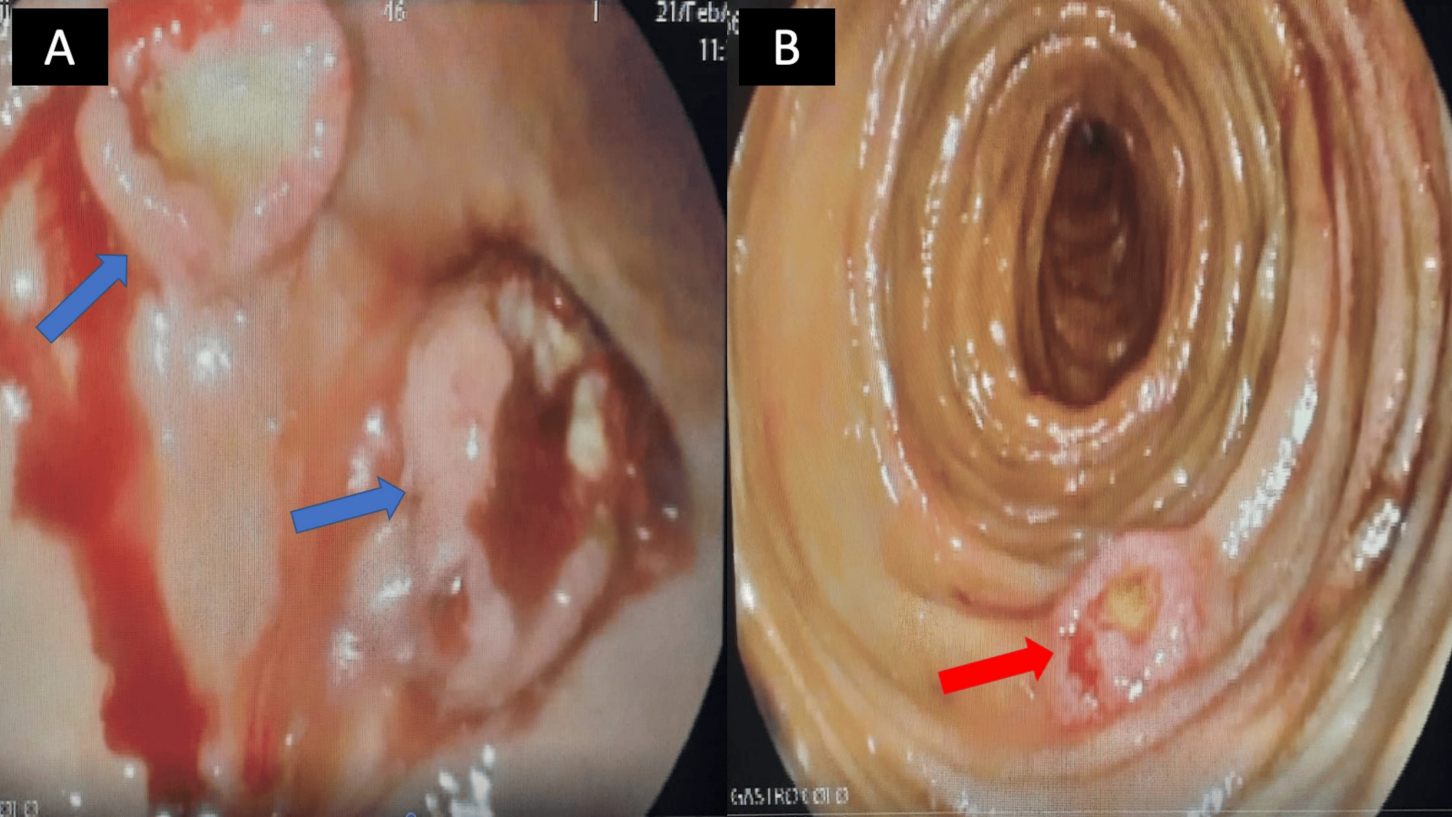
Gastroscopy findings (A) Ulcerated mass lesions in the gastric body (blue arrows). (B) Ulcerated mass lesions in segment 2 of the duodenum (red arrow).

The patient was admitted to the intensive care unit. She was started on omeprazole 40 mg twice daily and dexamethasone 4 mg every 8 hours intravenously (IV), and received 2 units of packed red blood cells. She underwent gastroscopy and colonoscopy 20 hours post-presentation. Colonoscopy findings were normal. Gastroscopy showed multiple fungating, ulcerating mass lesions in the stomach body (greater curvature) (Figure 1A). Lesions were friable and inflammatory with a central oozing ulceration. The surrounding mucosa was erythematous and edematous. In addition, similar lesions were also seen in segments 2 and 3 of the duodenum (Figure 1B).

Multiple biopsies were taken from the lesions seen. As endoscopic treatment, adrenaline injections were given inside and around the gastric and duodenal lesions. During hospitalization, hemoglobin levels returned to a baseline of 8.9 g/dL without any further drop, and melena was not seen again.

**Figure 2:**
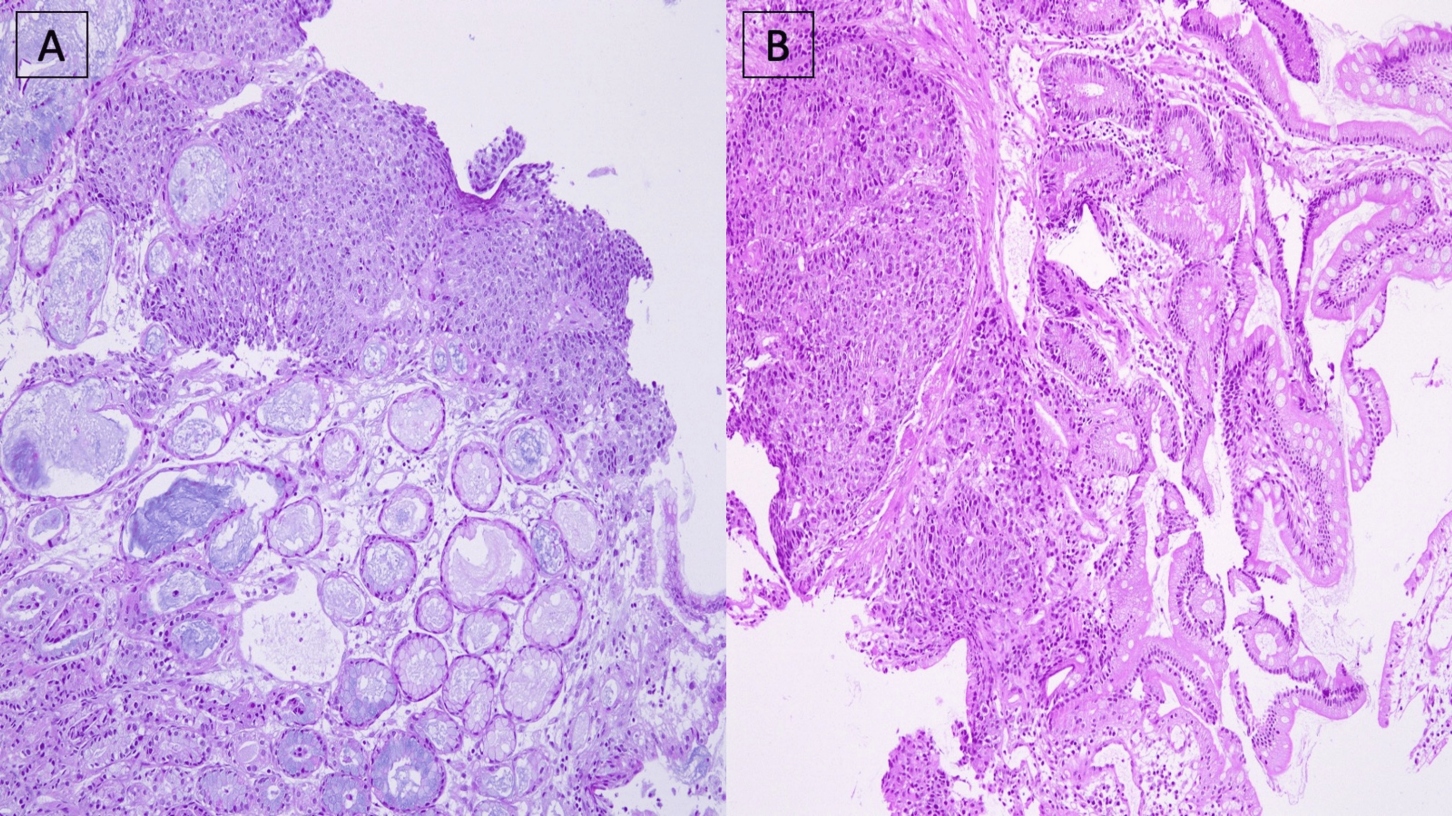
Histological findings Endoscopic biopsies stained with hematoxylin and eosin (H&E) with x100 magnification showing the presence of malignant squamous cells in the stomach (A) and duodenum (B).

On histopathology, biopsies taken from stomach lesions showed metastatic, moderately differentiated epidermoid carcinoma infiltrating the gastric mucosa. Biopsies taken from the duodenal lesions showed moderately differentiated epidermoid carcinoma infiltrating the intestinal submucosa (Figure 2).

These findings are consistent with metastasis from the cervical squamous cell carcinoma to the stomach and duodenum. The addition of Paclitaxel 100mg/m^2 ^to the chemotherapy regimen was planned to be offered, but the patient passed away one week after admission. 

## Discussion

Metastases to the gastrointestinal tract are infrequent, mostly occurring at a late stage of the malignant disease. Advances in cancer treatments resulted in extended patient survival and, therefore, a higher incidence of metastases to uncommon sites including the gastrointestinal tract [8]. Primary tumors metastasizing to the gastrointestinal tract are breast (38%), kidney (13%), lung (12%), prostate (8%), and ovary (7%) tumors [9]. The stomach is the main site of metastasis, accounting for 34% of the cases in the study by Rosty et al. and 63.2% in the study by Haendchen Bento et al. [9,10]. On the other hand, duodenal involvement is found in 13% to 29.5% of the cases. Metastasis of primary cervical malignancy to the gastrointestinal tract is among the least frequently observed. In addition, the concomitant occurrence of gastric and duodenal metastatic lesions encountered in our patient has not been previously reported.

The main pathway involved in cervical cancer metastasis is direct local tumor invasion to the vagina, bladder, and rectum. Other pathways include peritoneal dissemination, and hematogenous and lymphatic spread. Metastasis to distant organs, including the gastrointestinal tract, mainly follows the lymphatic dissemination via the para-aortic and mesenteric lymph nodes [11]. In addition, hematogenous spread is also possible, owing to the high vascularization of the gastric and bowel mucosa [12]. A study conducted by Zhou and Peng showed that single-site metastases (68.7%) are more frequent than metastasis to multiple organs. Moreover, the lungs are the most frequent distant site, accounting for 37.9% of the cases, followed by the bone (16.7%), the liver (12.5%), and the brain (1.6%) [13]. Hence, our patient has an atypical and infrequent presentation, with multiple metastases to distant organs including the lungs, bone, liver, stomach, and duodenum.

Few isolated cases of cervical squamous cell carcinoma metastasizing to the stomach are reported [14-21]. Squamous cell carcinoma was the predominant histopathological entity, similar to the case presented, except for one case of mucinous adenocarcinoma [19]. The chief complaints at presentation are various, mimicking primary gastric malignancy. These include dyspepsia [18,19], gastrointestinal bleed (hematemesis and melena) [16,17], anorexia [18], and dysphagia [21]. Non-specific symptoms such as epigastric or diffuse abdominal pain, nausea, vomiting, and fatigue may be present. Other cases are incidentally found on imaging by CT scan of the abdomen [19] and fluorodeoxyglucose-positron emission tomography (FDG-PET) scan [20]. On the other hand, only eight cases of duodenal metastatic lesions from a primary cervical malignancy have been reported [22-29]. Patients most commonly presented for upper gastrointestinal bleed [11,22], abdominal and epigastric pain [23-27], nausea and vomiting [24,25,28], and decreased weight and appetite [26]. Additionally, laboratory findings were consistent with anemia in all the reported cases. Our patient presented with gastrointestinal bleed and anemia, with a severe drop in hemoglobin (4.7 g/dL).

As recommended by the International Consensus Group, patients presenting with upper gastrointestinal bleed should undergo endoscopy within 24 hours of presentation. Endoscopic management should include thermocoagulation and sclerosant injection followed by proton-pump inhibitor therapy twice daily [29]. Our patient was put on omeprazole twice daily at admission, underwent endoscopy 18 hours following her presentation, and received epinephrine injections inside and around the lesions during endoscopy.

Endoscopic characteristics of metastatic tumors to the stomach are diverse, mimicking primary gastric cancer and therefore posing diagnostic challenges. Lesions are more commonly solitary (62.5%) than multiple (37.5%), primarily located in the upper and middle thirds of the stomach in 87.5% of the cases [30]. The findings in our patient are among the least common, as the lesions were multiple and located in both the stomach and the duodenum. The appearance of metastatic lesions is often characterized by small, polypoid, ulcerated masses. However, the lesions found in our patient were large in size.

Treatment options including chemotherapy regimens, radiotherapy, and palliative care depend on the primary malignancy and its staging. Nonetheless, surgical resection by gastrectomy remains the only potentially curative treatment [31]. In fact, the combination of cisplatin and paclitaxel is considered a standard regimen in cases of recurrent metastatic cervical cancer requiring palliative care [32]. However, prognosis remains poor despite optimal treatment since the presence of gastric metastasis is an indicator of advanced disease. In fact, 82.7% of patients having cervical squamous cell carcinoma with multiple metastasis died within one year, as the prognosis of multi-site metastasis is worse than single-site metastasis [33]. Additionally, the median survival period is three months from the diagnosis of gastric metastasis [34]. To note, our patient passed away one week following admission.

## Conclusions

Metastases to the gastrointestinal tract, specifically the stomach and duodenum, from primary cervical cancer are rare. However, a high index of suspicion should be kept in patients who are known to have cervical cancer, presenting with upper gastrointestinal bleed. In fact, metastases to the gastrointestinal tract are rare; however, they are encountered more frequently nowadays given the advances in cancer therapy. Although the prognosis is poor, early identification allows for adequate palliative care and symptomatic management.
